# Supplementary material for: Identification of a prismatic P3N3 molecule formed from electron irradiated phosphine-nitrogen ices
Source: Nat Commun. 2021 Sep 15;12:5467. doi: 10.1038/s41467-021-25775-1 (PMC8443655; doi:10.1038/s41467-021-25775-1)
Supplement: Supplementary file 1 — Supplementary information [file 41467_2021_25775_MOESM1_ESM.pdf]

Supplementary Information for

Identification of a prismatic P_3N_3 molecule formed from electron irradiated phosphine-nitrogen ices

Cheng Zhu[1,2], André K. Eckhardt[3*], Sankhabrata Chandra[1,2], Andrew M. Turner[1,2], Peter R. Schreiner[4], Ralf I. Kaiser[1,2*]

[1] Department of Chemistry, University of Hawaii at Manoa, 2545 McCarthy Mall, Honolulu, HI 96822 (USA)

[2] W. M. Keck Laboratory in Astrochemistry, University of Hawaii at Manoa, 2545 McCarthy Mall, Honolulu, HI 96822 (USA)

[3] Department of Chemistry, Massachusetts Institute of Technology, Cambridge, MA 02139 (USA)

[4] Institute of Organic Chemistry, Justus Liebig University, Heinrich-Buff-Ring 17, 35392 Giessen (Germany)

* Correspondence to: ake05@mit.edu, ralfk@hawaii.edu

This file includes:

Supplementary Figures 1 and 2

Supplementary Tables 1 to 4

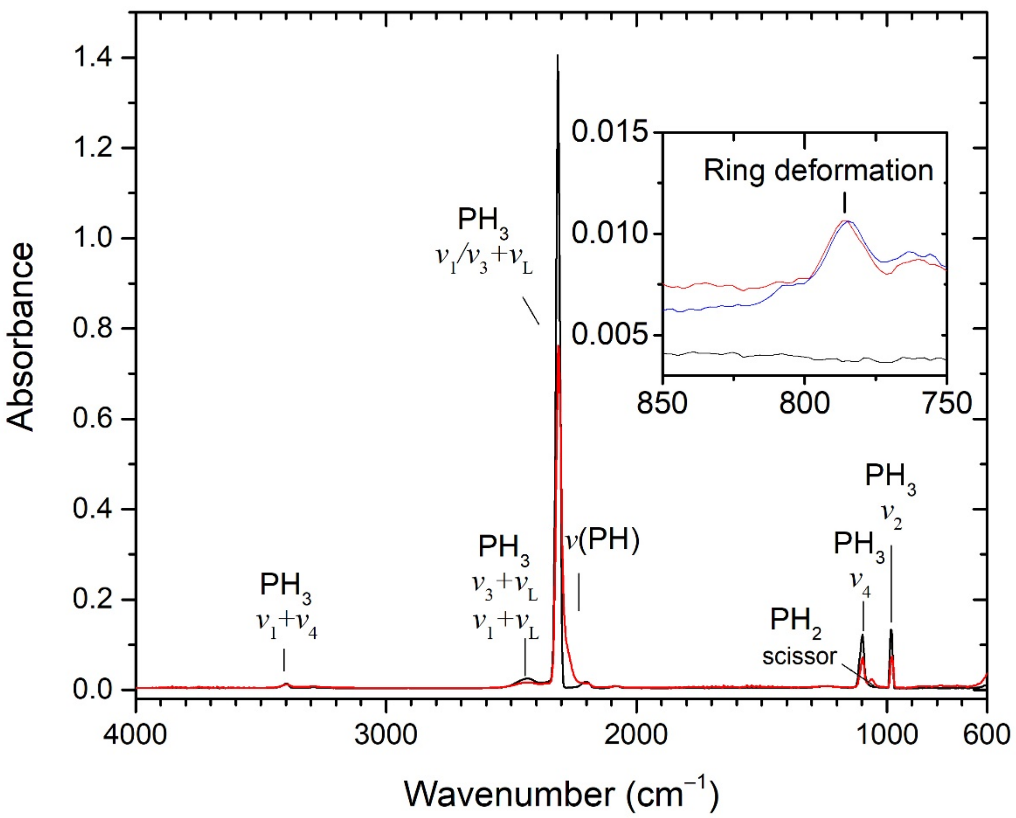

Supplementary Figure 1. FTIR spectra of the phosphine (PH_3) + nitrogen (N_2) ice before (black) and after (red) processing with energetic electrons. The inset expands the low-intensity region of the spectra and shows the FTIR spectrum of an electron processed phosphine (PH_3) + ^{15}N-nitrogen ($^{15}N_2$) ice (blue).

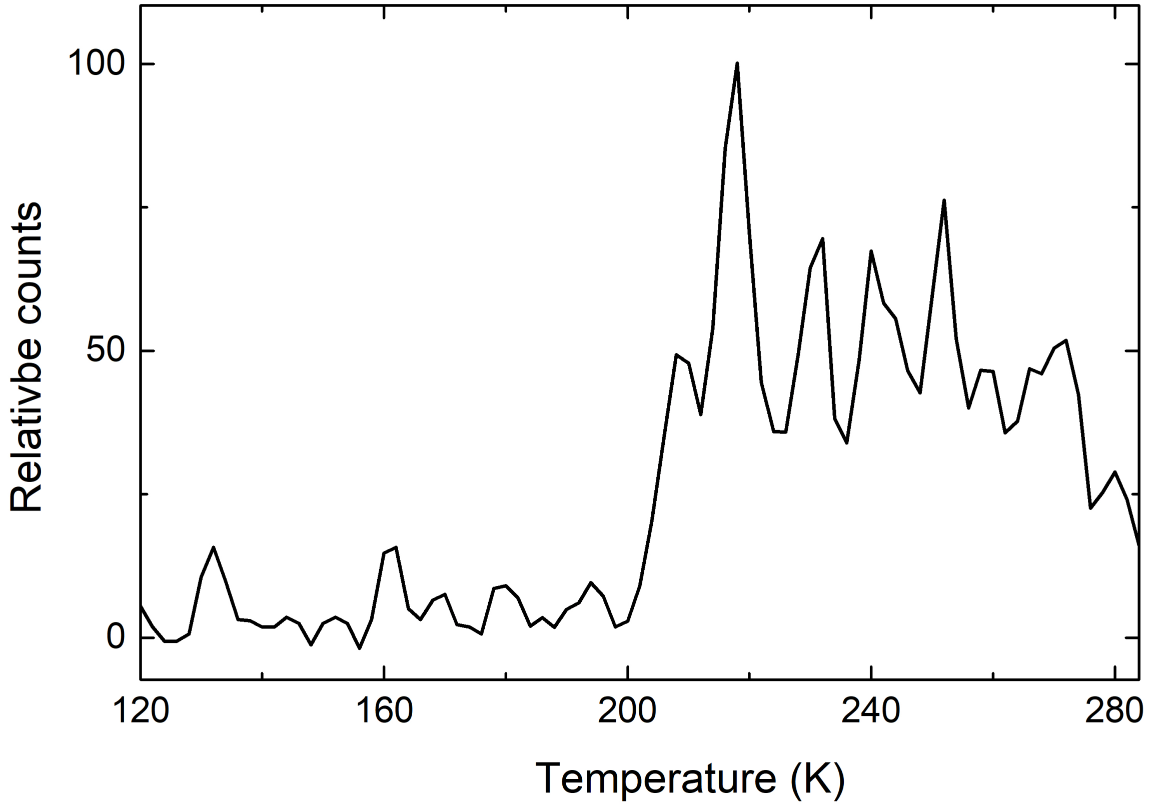

Supplementary Figure 2. Temperature programmed desorption (TPD) profile at *m/z* = 138. The data were recorded during the TPD phase of the electron processed phosphine (PH_3) + ^{15}N-nitrogen ($^{15}N_2$) ice via photoionization reflectron time-of-flight mass spectrometry (PI-ReTOF-MS) at a photon energy of 10.49 eV.

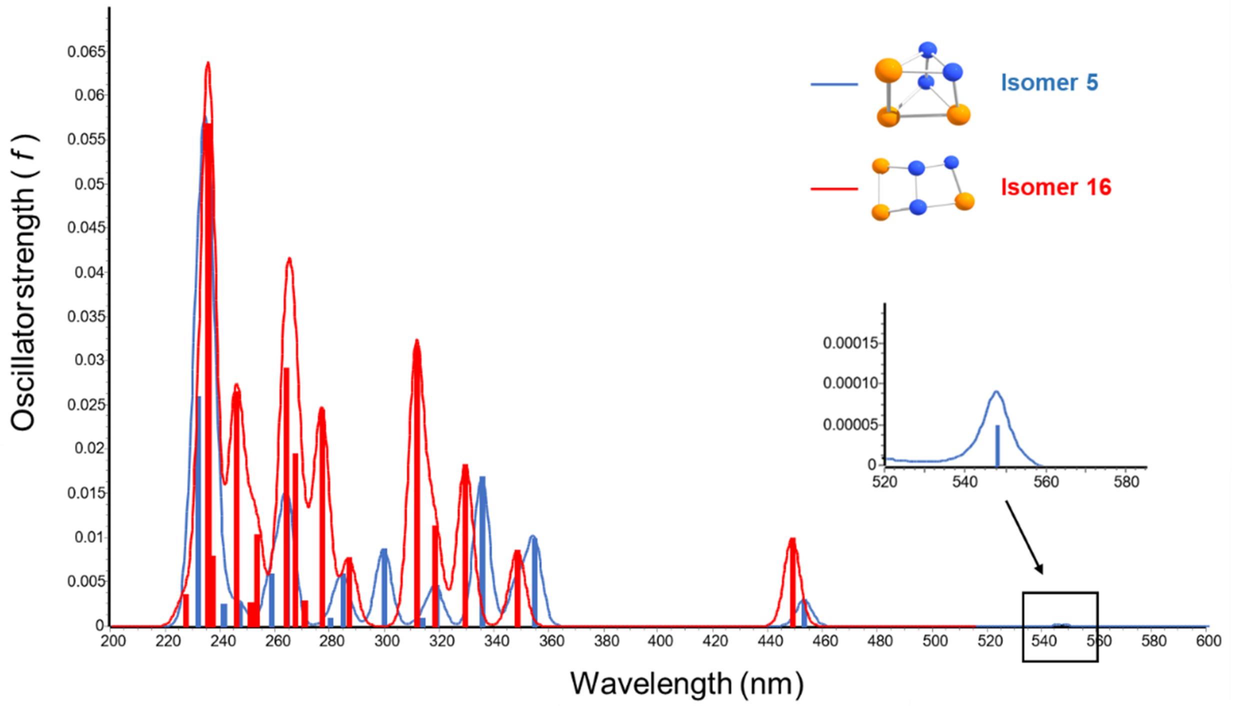

Supplementary Figure 3. Ultraviolet–visible (UV-Vis) spectra for P_3N_3 isomers 5 and 16. The spectra were computed at the TD-B3LYP/cc-pVTZ level of theory.

Supplementary Table 1. List of experiments.

#	Precursors	Electron current (nA)	Laser irradiation	Photoionization Energy (eV)
1	$PH_3 + N_2$	100	-	10.49
2	$PH_3 + {}^{15}N_2$	100	-	10.49
3	$PH_3 + N_2$	0 (blank)	-	10.49
4	$PH_3 + N_2$	100	-	8.53
5	$PH_3 + N_2$	100	-	8.47
6	$PH_3 + N_2$	100	547 nm	8.47
7	$PH_3 + N_2$	100	547 nm	8.75
8	$PH_3 + N_2$	100	-	8.20

Supplementary Table 2. Data used to calculate the average irradiation dose per molecule.

Initial kinetic energy of the electrons, E_{init} (keV)		5
Ice		$PH_3 + N_2$
Irradiation current, I (nA)		100 ± 5
Total number of electrons		$(4.5 \pm 0.5) \times 10^{15}$
Average penetration depth, l_{ave} (nm)[a]		490 ± 50
Maximum penetration depth, l_{max} (nm)[a]		880 ± 90
Average kinetic energy of backscattered electrons, E_{bs} (keV)[a]		2.96 ± 0.30
Fraction of backscattered electrons, f_{bs}[a]		0.12 ± 0.01
Average kinetic energy of transmitted electrons, E_{trans} (keV)[a]		0
Fraction of transmitted electrons, f_{trans}[a]		0
Irradiated area, A (cm^2)		1.0 ± 0.1
Dose (eV/molecule)	PH_3	26 ± 4
	N_2	21 ± 3

Note:

[a] Parameters obtained using the CASINO software v2.42.

Supplementary Table 3. Parameters for the vacuum ultraviolet (VUV) light generation used in the present experiments.[a]

$2\omega_1 - \omega_2$	Photoionization energy (eV)	10.49 ($3\omega_1$)	8.75	8.53	8.47	8.20
	Flux (10^{11} photons s^{-1})	12 ± 1	10 ± 1	10 ± 1	10 ± 1	10 ± 1
	Wavelength (nm)	118.222	141.696	145.351	146.380	151.200
ω_1	Wavelength (nm)	355	202.316	202.316	202.316	202.316
Nd:YAG (YAG A)	Wavelength (nm)	355	532	532	532	532
Dye laser (DYE A)	Wavelength (nm)	-	606.948	606.948	606.948	606.948
Dye		-	Rhodamine 610 and 640	Rhodamine 610 and 640	Rhodamine 610 and 640	Rhodamine 610 and 640
ω_2	Wavelength (nm)	-	353.5	332.5	327.5	305.5
Nd:YAG (YAG B)	Wavelength (nm)	-	532	532	532	532
Dye laser (DYE B)	Wavelength (nm)	-	707	665	655	611
Dye		-	LDS 722	DCM	DCM	Rhodamine 610 and 640
	Nonlinear medium	Xe	Kr	Kr	Kr	Kr

Note:

[a] The uncertainty for VUV photon energies is 0.001 nm.

Supplementary Table 4. Comparison of experimental to computed ionization energies (CCSD(T)/CBS//B3LYP/cc-pVTZ + zero-point vibrational energy (ZPVE) corrections) of different nitrogen- and phosphorus- containing compounds with average deviations computed from the error limits. Combined error limits are used to obtain the corrected computed ionization energies.

Compounds	Experimental IE (eV)	Experimental Error Limits (eV)	References	Computed IE (eV)	Computed IE – Experimental IE (max) (eV)	Computed IE – Experimental IE (min) (eV)
Ammonia NH_3	10.070 ± 0.020	10.050 - 10.090	1	10.15	+0.060	+0.100
Phosphine PH_3	9.869 ± 0.002	9.867 - 9.871	1	9.82	–0.051	–0.047
Hydrogen cyanide HCN	13.60 ± 0.01	13.59 - 13.61	1	13.57	–0.04	–0.02
Methinophosphide HCP	10.79 ± 0.01	10.78 - 10.80	2	10.76	–0.04	–0.02
Acetonitrile CH_3CN	12.20 ± 0.01	12.19 - 12.21	3	12.20	-0.01	+0.01
Methyl Isocyanide CH_3NC	11.24 ± 0.01	11.23 - 11.25	3	11.25	+0.00	+0.02
2H-Azirine c-H_2CCHN	10.05 ± 0.03	10.02 - 10.08	3	10.02	–0.06	+0.00
					Average –0.04 ± 0.04	Average 0.02 ± 0.05
					Error Limits –0.08 to +0.00	Error Limits –0.03 to +0.07
					Combined Error Limits –0.08 - +0.07	

Notes:

Reference 1: Lias (2018)[1].

Reference 2: Frost et al. (1973)[2].

Reference 3: Turner et al. (2021)[3].

Supplementary References

1 Sharon G. Lias, "Ionization Energy Evaluation" in NIST Chemistry WebBook, NIST Standard Reference Database Number 69, Eds. P.J. Linstrom and W.G. Mallard, National Institute of Standards and Technology, Gaithersburg MD, 20899, https://doi.org/10.18434/T4D303, **2018**.

2 Frost, D., Lee, S. & McDowell, C. The photoelectron spectrum of HCP and comments on the first photoelectron band of HCN. *Chem. Phys. Lett.* **23**, 472-475 (1973).

3 Turner, A. M., Chandra, S., Fortenberry, R. C. & Kaiser, R. I. A photoionization reflectron time-of-flight mass spectrometric study on the detection of ethynamine ($HCCNH_2$) and 2H-azirine (c-H_2CCHN). *ChemPhysChem* **22**, 985-994 (2021).
