# Supplementary material for: Identification of a prismatic P3N3 molecule formed from electron irradiated phosphine-nitrogen ices
Source: Nat Commun. 2021 Sep 15;12:5467. doi: 10.1038/s41467-021-25775-1 (PMC8443655; doi:10.1038/s41467-021-25775-1)
Supplement: Supplementary file 3 — Description of Additional Supplementary Files [file 41467_2021_25775_MOESM3_ESM.docx]

**Description of Additional Supplementary Files**

File Name: Supplementary Data 1

Description: **Supplementary Data 1.** Computed Cartesian coordinates (Å), vibrational frequencies (cm^−1^), and infrared (IR) intensities (km mol^−1^) for P_3_N_3_ isomers and the isomerization transition state between **5** and **16** (TS) at the B3LYP/cc-pVTZ level of theory

File Name: Supplementary Data 2

Description: **Supplementary Data 2.** Computed ultraviolet–visible (UV-Vis) absorptions and assignments for P_3_N_3_ isomers **5** and **16** at the TD-B3LYP/cc-pVTZ level of theory.
