# Supplementary material for: Identification of a prismatic P3N3 molecule formed from electron irradiated phosphine-nitrogen ices
Source: Nat Commun. 2021 Sep 15;12:5467. doi: 10.1038/s41467-021-25775-1 (PMC8443655; doi:10.1038/s41467-021-25775-1)
Supplement: Supplementary file 4 — Supplementary Data 1 [file 41467_2021_25775_MOESM4_ESM.zip › Supplementary Data 1.docx]

## **Supplementary Data 1.** Computed Cartesian coordinates (Å), vibrational frequencies (cm^−1^), and infrared (IR) intensities (km mol^−1^) for P_3_N_3_ isomers and the isomerization transition state between **5** and **16** (TS) at the B3LYP/cc-pVTZ level of theory.^a^

| 5  P 1.099540 –0.830542 –0.465917  P 0.664012 1.326123 –0.094510  N 0.712172 –0.030864 1.115255  N –0.668547 –1.208845 –0.299702  P –1.456325 0.365883 –0.314355  N –0.701968 –0.606287 1.058981  E = –1188.2985186  ZPVE = 9.9108 kcal mol^–1^  Freq Int  252.2129 1.4485  361.3209 1.1241  393.5096 3.7851  403.9446 6.9874  521.7742 15.2759  573.2704 0.2708  580.3031 4.9803  667.4761 13.155  691.1102 19.2057  748.9012 1.0949  824.0967 16.2899  914.775 18.4743 | 10  P –0.516335 –0.755606 1.112648  P –0.516335 –0.755606 –1.112648  N 0.872845 –0.891431 0.000000  N –0.516335 1.071785 0.737048  N –0.516335 1.071785 –0.737048  P 1.107256 0.926881 –0.000000  E = –1188.3465917  ZPVE = 9.8814 kcal mol^–1^  Freq Int  285.8673 2.2232  390.6837 1.1512  396.5698 1.9586  489.4323 7.116  522.187 8.6008  561.1532 4.9089  576.1047 65.0081  644.257 53.6133  659.4045 1.0947  698.6979 1.9744  753.4406 36.5263  934.3496 36.662 |
| --- | --- |
| 11  P –0.273295 1.161239 –0.382364  P –1.405613 –0.140896 1.032622  P –1.088787 –0.864559 –0.839681  N 1.451645 0.951477 0.016826  N 2.506541 –1.137326 0.253518  N 1.972591 –0.147975 0.135564  E = –1188.4098827  ZPVE = 10.5981 kcal mol^–1^  Freq Int  22.3008 0.0281  99.3332 0.0604  193.1723 0.3465  267.4196 2.993  394.027 1.1052  441.144 6.3782  527.7874 104.8406  589.3002 6.1876  647.0515 1.0118  684.8606 26.8309  1319.0089 187.6669  2228.0498 590.0207 | 12 (CS)  N –0.752986 1.297323 0.751880  P 1.241171 –1.068200 0.000000  N –1.440699 0.243476 0.000000  N –0.752986 1.297323 –0.751880  P 0.886928 1.165396 0.000000  P –0.752986 –1.421653 0.000000  E = –1188.2963432  ZPVE = 9.3287 kcal mol^–1^  Freq Int  175.9508 2.431  332.7307 3.6619  334.0225 3.946  386.4683 5.3546  478.0085 2.0769  499.1469 28.213  524.0386 0.2994  612.658 9.7516  642.4376 1.6736  671.7086 14.3015  794.9342 4.2678  1073.4234 1.9326 |
| 13  P 0.000000 1.304800 –0.576987  P 1.129990 –0.652400 –0.576987  P –1.129990 –0.652400 –0.576987  N 0.000000 0.848851 1.236401  N 0.735126 –0.424425 1.236401  N –0.735127 –0.424425 1.236401  E = –1188.2790659  ZPVE = 9.9934 kcal mol^–1^  Freq Int  334.8716 1.3941  334.8716 1.3949  363.1893 0  407.8567 1.7388  407.8567 1.7389  518.7158 0.0145  634.4847 10.9355  690.0043 3.0765  690.0043 3.0765  738.4848 2.7421  738.4848 2.7418  1131.6382 1.122 | 15  N –0.359709 1.176657 1.136664  P 0.885819 –0.888156 0.000000  N –0.359709 1.176657 –1.136664  N 0.362845 0.951058 –0.000000  P –0.359709 –0.326942 1.802224  P –0.359709 –0.326942 –1.802224  E = –1188.3321125  ZPVE = 10.0626 kcal mol^–1^  Freq Int  181.0754 3.0449  248.8169 7.2576  260.7134 6.5753  399.9894 1.7294  410.2338 13.293  512.8478 3.7438  575.0095 10.289  627.1942 12.188  891.1511 26.3274  909.5928 8.6507  992.9812 4.0616  1029.3122 34.4821 |
| 19  P –0.000000 1.467461 0.409284  P –0.000000 –1.467461 0.409284  N 0.000000 0.000000 –1.993381  N 0.000000 0.000000 –0.757788  P –0.000000 0.000000 1.920094  N 0.000000 0.000000 –3.117391  E = –1188.3614459  ZPVE = 10.5465 kcal mol^–1^  Freq Int  29.9382 0.0134  140.4695 0.9066  256.6549 0.394  310.8738 12.6527  398.464 14.8418  414.9921 0.0941  488.45 15.3359  524.4575 5.6134  543.5989 0.5178  774.2134 5.1385  1253.9394 245.8832  2241.3341 834.0181 | 21  P –0.437370 –1.666317 0.000000  N 1.382292 0.201120 0.000000  N –0.714147 1.160435 0.000000  N 0.000000 0.140785 0.000000  P 1.684963 –1.459235 0.000000  P –1.559395 2.424459 0.000000  E = –1188.3201865  ZPVE = 10.3628 kcal mol^–1^  Freq Int  87.2878 1.7805  109.9106 1.9747  336.4289 10.6877  351.3407 0.8226  420.7918 0.2269  436.6721 5.7297  518.0834 24.7661  618.1832 12.9865  809.1116 48.6666  940.0062 34.6394  988.9833 12.0788  1632.1164 631.4488 |
| TS  H5 –1.309571 0.669822 –0.401389  H5 1.321385 0.969645 –0.113132  N –0.274939 0.738496 0.948909  N –1.166735 –1.003019 –0.237792  H5 0.795351 –1.168140 –0.276614  N –0.307513 –0.764047 1.012124  E = –1188.2602828  ZPVE = 9.1650 kcal mol^–1^  *ν*_i_ = 943.9 cm^–1^ |  |

**Note:**

^a^ The data for isomers not shown here can be found in the work of Zhu, C. *et al.* The elusive cyclotriphosphazene molecule and its Dewar benzene–type valence isomer (P_3_N_3_). *Sci. Adv.* **6**, eaba6934 (2020).
