# Supplementary material for: Identification of a prismatic P3N3 molecule formed from electron irradiated phosphine-nitrogen ices
Source: Nat Commun. 2021 Sep 15;12:5467. doi: 10.1038/s41467-021-25775-1 (PMC8443655; doi:10.1038/s41467-021-25775-1)
Supplement: Supplementary file 5 — Supplementary Data 2 [file 41467_2021_25775_MOESM5_ESM.zip › Supplementary Data 2.docx]

**Supplementary Data 2**. Computed ultraviolet–visible (UV-Vis) absorptions and assignments for P_3_N_3_ isomers **5** and **16** at the TD-B3LYP/cc-pVTZ level of theory.

| Isomer | Wavelength (nm) | Oscillator strength (*f*) | Transition assignment | Orbital transitions |
| --- | --- | --- | --- | --- |
| **5** | 547.24 | 0.0001 | HOMO → LUMO  HOMO → LUMO+1 | 33 –> 34 (0.67513)  33 –> 35 (0.20098) |
|  | 453.27 | 0.0029 | HOMO–1 → LUMO  HOMO → LUMO  HOMO → LUMO+1 | 32 –> 34 (0.15854)  33 –> 34 (–0.18537)  33 –> 35 (0.65773) |
|  | 354.65 | 0.0097 | HOMO–1 → LUMO  HOMO–1 → LUMO+1  HOMO → LUMO+2 | 32 –> 34 (0.54255)  32 –> 35 (–0.32636)  33 –> 36 (0.27551) |
|  | 348.35 | 0.005 | HOMO–2 → LUMO  HOMO–1 → LUMO+1  HOMO → LUMO+2 | 31 –> 34 (0.10225)  32 –> 35 (0.49046)  33 –> 36 (0.48561) |
|  | 335.52 | 0.0168 | HOMO–1 → LUMO  HOMO–1 → LUMO+1  HOMO → LUMO+1  HOMO → LUMO+2 | 32 –> 34 (–0.39539)  32 –> 35 (–0.37683)  33 –> 35 (0.12166)  33 –> 36 (0.40396) |
|  | 318.67 | 0.0044 | HOMO–3 → LUMO  HOMO–2 → LUMO  HOMO–2 → LUMO+1 | 30 –> 34 (0.36579)  31 –> 34 (0.52406)  31 –> 35 (0.26908) |
|  | 313.85 | 0.0008 | HOMO–3 → LUMO  HOMO–2 → LUMO  HOMO–2 → LUMO+1 | 30 –> 34 (0.59793)  31 –> 34 (–0.30631)  31 –> 35 (–0.18929) |
|  | 299.72 | 0.0087 | HOMO–3 → LUMO+1  HOMO–2 → LUMO  HOMO–2 → LUMO+1 | 30 –> 35 (0.16961)  31 –> 34 (–0.28054)  31 –> 35 (0.60383) |
|  | 284.71 | 0.0057 | HOMO–3 → LUMO+1  HOMO–2 → LUMO  HOMO–2 → LUMO+1 | 30 –> 35 (0.67005)  31 –> 34 (0.10305)  31 –> 35 (–0.12315) |
|  | 280.16 | 0.0008 | HOMO → LUMO+3 | 33 –> 37 (0.69097) |
|  | 264.47 | 0.0143 | HOMO–1 → LUMO+2 | 32 –> 36 (0.68037) |
|  | 258.8 | 0.0057 | HOMO–4 → LUMO  HOMO–1 → LUMO+2  HOMO → LUMO+4 | 29 –> 34 (0.67486)  32 –> 36 (–0.10204)  33 –> 38 (0.10256) |
|  | 246.96 | 0.0025 | HOMO–4 → LUMO+1  HOMO–2 → LUMO+2  HOMO → LUMO+4 | 29 –> 35 (0.60286)  31 –> 36 (–0.20406)  33 –> 38 (–0.27935) |
|  | 241.19 | 0.0023 | HOMO–4 → LUMO+1  HOMO–3 → LUMO+2  HOMO–2 → LUMO+2 | 29 –> 35 (0.17796)  30 –> 36 (0.44518)  31 –> 36 (0.48872) |
|  | 235.66 | 0.0456 | HOMO–5 → LUMO+1  HOMO–4 → LUMO+1  HOMO–3 → LUMO+2  HOMO–2 → LUMO+2  HOMO → LUMO+4 | 28 –> 35 (–0.13790)  29 –> 35 (0.23814)  30 –> 36 (0.18438)  31 –> 36 (–0.16459)  33 –> 38 (0.55684) |
|  | 231.61 | 0.0258 | HOMO–4 → LUMO+1  HOMO–3 → LUMO+2  HOMO–2 → LUMO+2  HOMO–1 → LUMO+3  HOMO → LUMO+4 | 29 –> 35 (–0.16098)  30 –> 36 (0.49758)  31 –> 36 (–0.38880)  32 –> 37 (0.11653)  33 –> 38 (–0.15020) |
| **16** | 448.88 | 0.0099 | HOMO–2 → LUMO  HOMO → LUMO | 31 → 34 (0.12564)  33 → 34 (0.68460) |
|  | 348.53 | 0.0085 | HOMO–1 → LUMO  HOMO → LUMO+1  HOMO → LUMO+2 | 32 → 34 (–0.42401)  33 → 35 (0.52992)  33 → 36 (0.15625) |
|  | 329.61 | 0.0182 | HOMO–2 → LUMO  HOMO–1 → LUMO  HOMO → LUMO+1  HOMO → LUMO+2 | 31 → 34 (0.29202)  32 → 34 (0.32504)  33 → 35 (0.12594)  33 → 36 (0.52937) |
|  | 318.32 | 0.0113 | HOMO–2 → LUMO  HOMO–1 → LUMO+1  HOMO → LUMO+1  HOMO → LUMO+2 | 31 → 34 (0.53945)  32 → 35 (0.14128)  33 → 35 (0.15648)  33 → 36 (–0.37721) |
|  | 311.74 | 0.0315 | HOMO–3 → LUMO  HOMO–2 → LUMO  HOMO–2 → LUMO+1  HOMO–1 → LUMO  HOMO → LUMO+1  HOMO → LUMO+2  HOMO → LUMO+3 | 30 → 34 (–0.13701)  31 → 34 (–0.26824)  31 → 35 (0.15583)  32 → 34 (0.35850)  33 → 35 (0.34127)  33 → 36 (–0.19004)  33 → 37 (0.27530) |
|  | 287.01 | 0.0076 | HOMO–3 → LUMO  HOMO–1 → LUMO+1  HOMO–1 → LUMO+2  HOMO → LUMO+3 | 30 → 34 (0.10381)  32 → 35 (–0.22825)  32 → 36 (–0.39788)  33 → 37 (0.48126) |
|  | 277.1 | 0.0244 | HOMO–4 → LUMO  HOMO–3 → LUMO  HOMO–2 → LUMO+1  HOMO–1 → LUMO+1  HOMO–1 → LUMO+2  HOMO → LUMO+3 | 29 → 34 (0.26696)  30 → 34 (0.10573)  31 → 35 (–0.29945)  32 → 35 (0.41916)  32 → 36 (0.16390)  33 → 37 (0.30025) |
|  | 270.89 | 0.0027 | HOMO–4 → LUMO  HOMO–3 → LUMO  HOMO–2 → LUMO+1  HOMO–1 → LUMO+2  HOMO → LUMO+3 | 29 → 34 (0.45440)  30 → 34 (0.30012)  31 → 35 (0.12364)  32 → 36 (–0.34400)  33 → 37 (–0.19976) |
|  | 267.43 | 0.0194 | HOMO–4 → LUMO  HOMO–3 → LUMO  HOMO–2 → LUMO+1  HOMO–1 → LUMO+1  HOMO–1 → LUMO+2  HOMO → LUMO+1 | 29 → 34 (–0.38890)  30 → 34 (0.11891)  31 → 35 (0.10294)  32 → 35 (0.41827)  32 → 36 (–0.29071)  33 → 35 (–0.10224) |
|  | 264.06 | 0.0291 | HOMO–4 → LUMO  HOMO–3 → LUMO  HOMO–2 → LUMO+1  HOMO–1 → LUMO+1  HOMO–1 → LUMO+2  HOMO → LUMO+3 | 29 → 34 (–0.12623)  30 → 34 (0.51585)  31 → 35 (0.23859)  32 → 35 (–0.11202)  32 → 36 (0.30735)  33 → 37 (0.11881) |
|  | 253.28 | 0.0102 | HOMO–5 → LUMO  HOMO–2 → LUMO+2 | 28 → 34 (0.68554)  31 → 36 (–0.12773) |
|  | 251.1 | 0.0026 | HOMO–2 → LUMO+1  HOMO–2 → LUMO+2  HOMO–1 → LUMO+3  HOMO → LUMO+4 | 31 → 35 (0.11439)  31 → 36 (0.12504)  32 → 37 (0.62672)  33 → 38 (–0.23864) |
|  | 245.93 | 0.0264 | HOMO–3 → LUMO  HOMO–3 → LUMO+1  HOMO–2 → LUMO+1  HOMO–2 → LUMO+2  HOMO → LUMO+3  HOMO → LUMO+4 | 30 → 34 (–0.12574)  30 → 35 (–0.11967)  31 → 35 (0.37778)  31 → 36 (0.41539)  33 → 37 (0.11256)  33 → 38 (0.29076) |
|  | 236.96 | 0.0078 | HOMO–4 → LUMO+1  HOMO–2 → LUMO+2  HOMO–2 → LUMO+3  HOMO–1 → LUMO+1  HOMO–1 → LUMO+3  HOMO → LUMO+4 | 29 → 35 (0.11023)  31 → 36 (–0.26147)  31 → 37 (0.12173)  32 → 35 (0.10512)  32 → 37 (0.28448)  33 → 38 (0.52498) |
|  | 235.43 | 0.0568 | HOMO–4 → LUMO  HOMO–4 → LUMO+1  HOMO–3 → LUMO  HOMO–3 → LUMO+2  HOMO–2 → LUMO+1  HOMO–2 → LUMO+2  HOMO–2 → LUMO+3  HOMO–1 → LUMO  HOMO–1 → LUMO+1  HOMO–1 → LUMO+4  HOMO → LUMO+1  HOMO → LUMO+4 | 29 → 34 (–0.14929)  29 → 35 (–0.15352)  30 → 34 (0.13138)  30 → 36 (–0.14441)  31 → 35 (–0.30914)  31 → 36 (0.41152)  31 → 37 (0.10817)  32 → 34 (0.12432)  32 → 35 (–0.11598)  32 → 38 (0.12333)  33 → 35 (0.13918)  33 → 38 (0.14022) |
